# Affect regulation in the context of sexual and gender minority stress: A scoping review protocol

**DOI:** 10.1371/journal.pone.0329531

**Published:** 2026-01-05

**Authors:** Daphne Y. Liu, Benjamin A. Swerdlow, Shao Yuan Chong, Nadia Kako, Alex Rubin, Nicholas S. Perry

**Affiliations:** 1 Department of Psychology, University of Denver, Denver, Colorado, United States of America; 2 Department of Psychology, Lake Forest College, Lake Forest, Illinois, United States of America; Faculty of Medicine Vajira Hospital, Navamindradhiraj University, THAILAND

## Abstract

**Objective:**

To provide a broad, comprehensive picture of affect regulation in the context of sexual and gender minority stress, this scoping review aims to identify and synthesize methods, methodologies, and available evidence pertinent to emotion regulation and coping in the context of minority stress among sexual and gender minority (SGM) people.

**Introduction:**

SGM people face disproportionately high rates of mental health problems due to experiences of minority stress and lack of social safety. Theories and growing evidence suggest that affect regulation plays a critical role in SGM people’s well-being in the face of minority stress. Researchers have largely studied emotion regulation, coping, and minority stress in distinct literatures; as such, there is a critical need to synthesize evidence across these bodies of research.

**Inclusion criteria:**

We will review empirical studies that (1) included SGM people, (2) measured at least one affect regulation construct, and (3) studied affect regulation in the context of sexual or gender minority stress.

**Methods:**

Published and unpublished (i.e., grey literature) empirical studies written in English (no restrictions on publication date) will be searched using the following databases: PsycINFO (via EBSCO), Web of Science Core Collection (via Clarivate), PubMed (via National Library of Medicine), Gender Studies Database (via EBSCO), Sociological Abstracts (via ProQuest), and SocIndex with Full Text (via EBSCO). Grey literature will be identified through searching on additional repositories and databases and emailing listservs of relevant organizations. Potentially relevant papers will first be screened based on title and abstract, followed by full-text screening, against inclusion criteria by two independent reviewers. Data on study characteristics and findings relevant to the review will be extracted by two independent reviewers. Descriptive data relevant to each research question will be presented in tabular format, followed by a narrative summary of main findings, research gaps, and areas for future research.

## Introduction

Sexual and gender minority (SGM) people—individuals who do not identify as heterosexual or whose gender identity does not align with their sex assigned at birth [1–3]—are at disproportionately high risk for mental health problems (e.g., depression, anxiety, suicide attempts) [4,5]. The elevated rates of psychological problems reported by SGM people can be partly attributed to experiences of discrimination, prejudice, and stigma based on their minoritized sexual and/or gender identities, which are collectively known as sexual and gender minority stress (hereafter *minority stress*) [6–10]. Minority stressors exist at both the distal level (i.e., external, objective stressors, such as discriminatory hiring practices or enacted social rejection) and the proximal level (i.e., internalized stressors that are theorized to result from exposure to external stressors, such as identity-related shame or expectations of rejection) [7,8,11]. The lack of social safety (i.e., reliable social connections, social inclusion, and social protection) that SGM people experience in their social environments is also thought to negatively impact their mental health [12].

The root causes of minority stress undoubtedly exist at the societal and institutional levels and require systemic interventions [13]. Nonetheless, the adverse affective experiences SGM people endure as a result of exposure to minority stressors can have both short- and long-term negative impacts on the physical and mental health of individuals; as such, there may be opportunities for individuals to engage in effective and context-appropriate coping and emotion regulation to mitigate these negative consequences [14,11]. *Coping* refers to a process that is initiated in response to stress and that involves “constantly changing cognitive and behavioral efforts to manage specific external and/or internal demands that are appraised as taxing or exceeding the resources of the person” (p. 141) [15]. *Emotion regulation* refers to “the processes by which individuals influence which emotions they have, when they have them, and how they experience and express these emotions” (p. 275) [16]. Although distinct in several important ways, coping and emotion regulation are both goal-directed processes that share significant conceptual and measurement similarities [17,18]. Thus, in line with other scholars [17,18], we use *affect regulation* as an umbrella term to refer to both coping and emotion regulation.

Growing theory and evidence suggest that SGM people’s affect regulation abilities and behaviors have critical implications for their psychological well-being in the face of minority stress [11,8, 19,20. For example, Meyer’s [8] minority stress model posits that individuals’ coping capacities can serve as an important moderator of the link between minority stress and mental health outcomes. Specifically, having access to and the awareness to use personal (e.g., self-acceptance) and group (e.g., an LGBTQ+ affirming church) coping resources may ameliorate and counteract the impact of minority stress on well-being. Additionally, certain proximal stressors, such as identity concealment, can potentially serve affect regulatory functions themselves in connection with anticipated distal stressors (e.g., concealing one’s sexual orientation to reduce the threat of antigay violence) [8], although use of these strategies may result in negative psychological and physical outcomes in the long term [8,21,22]. Hatzenbuehler’s [11] psychological mediation framework presents an alternative view of the role of affect regulation in SGM people. Specifically, according to the psychological mediation framework, coping and emotion regulation are general psychological processes that serve as mediating mechanisms through which minority stress contributes to health disparities. As discussed by Hatzenbuehler [11], exposure to minority stress not only prompts affect regulation efforts that confer risk for psychopathology (e.g., rumination, suppression), but it also poses substantial challenges for affect regulation (e.g., insofar as it may be resource-depleting or furnish relatively few affordances that could facilitate regulation). Further, repeated exposure to minority stress may have deleterious consequences for the ongoing development of affect regulation skills and habits. Thus, over time, exposure to minority stress may jeopardize one’s affect regulation resources and abilities, thereby increasing risk for mental health problems. There is increasing evidence supporting the theory that minority stress increases maladaptive affect regulation processes (e.g., rumination), which in turn contributes to psychopathology (e.g., depressive symptoms) [23,24]. Together, then, both theories and growing empirical research support the important role of affect regulation in SGM people’s well-being in the face of minority stress. In fact, many psychological interventions tailored for SGM populations focus on enhancing affect regulation skills [25–28].

One barrier to advancing our understanding of affect regulation in the context of minority stress is that coping and emotion regulation come from largely distinct literatures. Put differently—and notwithstanding acknowledgements in emotion regulation research of the contributions from the stress and coping literatures [29,30]—coping and emotion regulation have largely advanced as separate areas of research, with each having their own theoretical boundaries, frameworks, terminologies, and preferred measures [30,31]. For example, past literature reviews have often focused on either coping *or* emotion regulation [32,33]. In parallel with the development of the coping and emotion regulation literatures, separate lines of empirical research have been conducted to understand coping [34] and emotion regulation [35] in the minority stress context, despite the fact that coping and emotion regulation have often been treated as largely interchangeable in theoretical work on minority stress [11]. Given the substantial overlap between coping and emotion regulation and growing support for their relevance to the minority stress context and SGM people’s well-being, we argue that it is time to comprehensively synthesize the body of work that has focused on affect regulation in the minority stress context among SGM people.

As outlined above, minority stress theories and research have often drawn on constructs related to affect regulation. However, most of these affect regulation constructs were not developed for measuring emotion regulation or coping specifically in the context of minority stress context or based on SGM people’s lived experiences. Instead, researchers interested in studying affect regulation in the context of minority stress have commonly used more generalized affect regulation constructs and measures and applied them to minority stress [36–38]. On the one hand, coping and emotion regulation are general psychological processes that are applicable to many situations, contexts, and populations and have far-ranging implications for psychological well-being [11,39,40]. Thus, using the frameworks and measures from the general coping and emotion regulation literatures may well be appropriate. On the other hand, because minority stress represents a unique form of social stress that is salient to SGM people [8], it is unclear whether these general frameworks and measures fully capture the scope, nuances, and salient aspects of affect regulation SGM people engage in in response to minority stress (e.g., with respect to motives, tactics, etc.). Because of this, we think it is critical to evaluate the methodologies and methods researchers have used to assess affect regulation in the context of minority stress, as well as the extent to which researchers have taken a more bottom-up approach to studying affect regulation in the minority stress context based on SGM people’s lived experiences.

Taken together, we propose to conduct a scoping review to provide a broad, comprehensive picture of affect regulation in the minority stress context. The **objective** of this scoping review is to identify and synthesize methods, methodologies, and available evidence pertinent to affect regulation in the context of minority stress among SGM people. In order for a study to have examined “affect regulation in the context of minority stress,” the study needs to have (a) measured affect regulation that takes place specifically in response to experienced or anticipated minority stress, (b) experimentally induced minority stress while studying affect regulation, *or* (c) examined the interplay between affect regulation and minority stress, such as in moderation or mediation analyses (see Inclusion Criteria below for more details of the boundaries of this definition and examples that fall within and outside the scope of this review). We chose to conduct a scoping review because it is well-suited for comprehensively mapping research evidence on a topic, synthesizing broad literatures that have not been integrated, providing an understanding of the state of the literature, and identifying gaps [41,42]. A preliminary search of PsycINFO, PubMed, Google Scholar, the Cochrane Database of Systematic Reviews and JBI Evidence Synthesis was conducted, and no published or in-progress systematic reviews or scoping reviews on this topic were identified. A comprehensive review of the extant literature on affect regulation can not only provide a clear picture of how researchers have studied affect regulation in the minority stress context, but it can also identify future directions and recommendations for future work. This review is intended to encourage dialogue and collaboration among researchers across disciplines and guide future interdisciplinary collaborations. We also consider this scoping review to be an initial step for determining whether systematic reviews on certain topics within this broader integration are warranted.

### Review questions

This scoping review aims to address the following four research questions:

1. What kinds of research questions have researchers tried to answer when investigating affect regulation in the context of minority stress?
2. What research designs (e.g., cross-sectional or longitudinal; quantitative, qualitative, or mixed methods; experimental, quasi-experimental, or descriptive) and analyses (e.g., mediation, moderation, thematic analysis) have researchers used to address these questions?
3. How have researchers measured affect regulation in the context of minority stress (e.g., global self-report surveys, laboratory-based observations, daily diaries, ecological momentary assessment, qualitative interviews)?
4. To what extent have researchers attended to the experiences of subgroups among SGM populations or to intersecting identities of SGM people (e.g., SGM people of color)?

## Materials and methods

This scoping review protocol was developed and written according to guidelines proposed by the Joanna Briggs Institute (JBI) Methodology Group [43]. We included a completed checklist of preferred reporting items recommended for protocol reviews (see S1 Table) [43], which was adapted from the preferred reporting items for systematic review and meta-analysis protocols (PRISMA-P) [44]. This scoping review will be conducted in accordance with the *a priori* protocol developed based on JBI methodology for scoping reviews [42,45]. This protocol has been pre-registered on the Open Science Framework (OSF; https://osf.io/k456p). The results of this scoping review will be reported according to the Preferred Reporting Items for Systematic Reviews and Meta-Analysis: Extension for Scoping Reviews (PRISMA-ScR) [46]. Any deviations from the protocol will be reported in the Method section of the final publication following the completion of this review. This scoping review involves reviewing published and grey literature and thus does not require Institutional Review Board review and approval. We will not have access to data that could identify participants of reviewed studies.

### Inclusion criteria

The inclusion criteria of this scoping review are organized based on JBI’s Population, Concept, and Context (PCC) framework [43].

### Participants

**All, or a subsample**, **of participants of the included studies need to have identified themselves as SGM**. Participants may be of any age, and there is no specified minimum number or percent of the sample that needs to have identified as SGM. However, studies are excluded if no participants identified as SGM, or if the authors did not report participants’ sexual or gender identity.

### Concept

The study needs to have assessed **at least one affect regulation construct**. Affect regulation encompasses emotion regulation and coping. The affect regulation construct may be related to a person’s general affect regulation ability or to their use of specific affect regulation strategies or behaviors (e.g., the tendency to engage in cognitive reappraisal). The study may have assessed affect regulation or coping using any method, and data may be quantitative or qualitative. In order for the study to meet the Concept inclusion criterion, the study methods need to have included some direct assessment of affect regulation (i.e., the concept must be evident in the methods the researchers have used, not just in their framing of the study or results). Some examples of this include: (a) using one or more measures that are designed to assess people’s affect regulation beliefs, tendencies, abilities, strategies, or tactics; (b) explicitly asking participants how they regulated their affect in a particular instance; or (c) experimentally instructing participants to regulate their affect or observing how participants regulated affect in response to a stimulus.

Of note, many behaviors can be conceptualized as having affect-regulating features in at least some cases (e.g., substance use, social support). For the purpose of this scoping review, it must be clear from the paper that the assessed construct was explicitly measured *as an affect regulation strategy*; in other words, simply conceptualizing the construct as an affect regulation strategy (e.g., describing substance use as sometimes being motivated by coping goals in the Introduction section of the publication) is not sufficient. For example, a paper that used the substance use subscale of the COPE Inventory (e.g., “I use alcohol or drugs *to make myself feel better*” [emphasis added]) would likely qualify for this inclusion criterion, whereas a paper that used a generic measure of substance use frequency (“In the past week, how many times have you used tobacco?”) might not qualify even if the authors included some discussion of the possibility that tobacco use might be prompted by a desire to reduce stress in some cases. This is because, by definition, affect regulation is a goal-directed process that involves the activation of a goal to regulate affect and requires a person’s active efforts to manage internal or external demands or influence emotions [30]. This effort may come from oneself (e.g., an SGM person suppresses their emotional expressions when anticipating discrimination) or from other people (e.g., an SGM person’s spouse verbalizes affirmations to help the person cope with internalized stigma). Cases, though, where it is not clear at the level of research methods that the behavior is related to affect regulation motives or goals, would not meet inclusion criteria. In a similar vein, whereas perceived affect regulation abilities and behaviors are acceptable constructs of affect regulation (e.g., subscale or total scores on the Difficulties in Emotion Regulation Scale) [47], measures of beliefs and perceptions that are not specifically and directly related to people’s affect regulation goals or efforts would not meet the inclusion criteria of concept even if those beliefs and perceptions might have downstream consequences for affective experience or affect regulation (e.g., perceived availability of general social support, such as a person indicating that there are people in their life who would be willing to help them move or to pick them up from the airport).

### Context

Included studies need to have studied affect regulation **in the context of sexual or gender minority stress**. The spirit of this inclusion criterion is to ensure that we are including research that is at the *intersection* between affect regulation and minority stress, rather than studying these two constructs separately without directly connecting them. In line with this goal, we delineate several ways in which the study can meet the Context inclusion criterion. The most straightforward case would be researchers including questions or scale items that explicitly asked participants about their affect regulation related to minority stress experiences (e.g., “Which of the following behaviors do you typically engage in to cope with experiences of discrimination related to your sexual orientation?”). A second case would be researchers experimentally inducing a minority stress context and then instructing participants to engage in affect regulation or assessed participants’ spontaneous engagement in affect regulation (e.g., exposing SGM participants to passages containing homophobic content and asking them to engage in cognitive reappraisal to improve their affect). A third case would be researchers directly examining the association of affect regulation with minority stress in their data analyses, such as through mediation or moderation analyses. In the case of mediation, the authors could have examined whether rumination (an emotion regulation strategy) explains the association between recent experiences with gender-based discrimination and current depressive symptoms by testing the following mediation in line with the psychological mediation framework [11]: discrimination → rumination → depressive symptoms. In the case of moderation, the authors could have examined either how affect regulation moderates the link between minority stress and an outcome (e.g., test whether active coping styles attenuate the association between internalized transphobia and psychological distress) or how minority stress moderates the link between affect regulation and an outcome (e.g., test whether using alcohol to cope is more strongly associated with subsequent depression for those who experience more frequent discrimination).

On the other hand, a study would be excluded if the researchers did not do any of the following: (1) assess affect regulation specifically in response to experienced or anticipated minority stress, (2) experimentally induce minority stress while studying affect regulation, or (3) examine the interplay between affect regulation and minority stress in mediation or moderation analyses. We describe two example scenarios in which the Context inclusion criterion is *not* met. One scenario would be a study simply examining the bivariate association between general affect regulation and minority stress with no clear indication that affect regulation takes place in response to minority stress (e.g., testing whether people who more frequently use acceptance as an emotion regulation strategy tend to report lower internalized homophobia). A second scenario that would not meet this inclusion criterion would be a study examining how general affect regulation and minority stress constructs are separately associated with other constructs in the study, again with no clear indication that affect regulation takes place in response to minority stress. For example, a study that examined how passive coping and identity-related self-stigma independently predict depressive symptoms one month later by including passive coping and self-stigma simultaneously as predictors in a regression model (without examining their interaction with each other) would fall outside the scope of this review.

### Types of evidence sources

This review will include published and unpublished research papers that feature one or more empirical studies, defined as studies that derive scientific evidence based on concrete data collected from participants. We will include cross-sectional and longitudinal studies, studies that collect quantitative or qualitative data, and studies using a variety of methods for assessing affect regulation (e.g., surveys, laboratory-based experiments, experience sampling methods, qualitative interviews). We will exclude books, book chapters, reviews, opinion papers, commentaries, and protocol papers. We will limit our review to only papers written in English given that all authors are trained in conducting research in English.

### Search strategy

The search strategy will aim to locate published and unpublished studies that meet our inclusion criteria. This strategy was developed collectively by the authors, in consultation with an experienced research librarian at the University of Denver. For the sake of comprehensiveness, we will not have restrictions on publication dates. Specific databases were identified based on disciplinary relevance (e.g., databases that researchers have used in past reviews on related topics): PsycINFO (via EBSCO), Web of Science Core Collection (via Clarivate), PubMed (via National Library of Medicine), Gender Studies Database (via EBSCO), Sociological Abstracts (via ProQuest), and SocIndex with Full Text (via EBSCO). We plan to conduct an initial round of searches of these databases at the beginning of our scoping review process and then to conduct updated searches approximately six months prior to the anticipated date of submitting the resulting manuscript for peer review to capture additional research that becomes available after the initial search. Grey literature will be additionally searched via the following sources or methods: (a) Google Scholar, (b) commonly used social sciences repositories (i.e., PsyArXiv, PsychArchives, OSF preprints, Social Science Research Network[SSRN]), (c) dissertation and thesis databases (i.e., ProQuest Dissertations & Theses Global, Open Access Theses and Dissertations WorldCat), and (d) emails soliciting unpublished research through relevant organizations’ listservs (i.e., Society for Affective Science, American Psychological Association Division 44: Society for the Psychology of Sexual Orientation and Gender Diversity, Association for Association for Behavioral and Cognitive Therapies).

A list of search terms pertaining to inclusion criteria was formulated. The search terms can be grouped into three categories: terms related to SGM populations (e.g., LGBT*, sexual and gender minorit*), terms related to affect regulation (e.g., *coping*, *emotion regulation*), and terms related to minority stress (e.g., *minority stress*, *discrimination*). These categories correspond to the inclusion criteria of participants, concept, and context, respectively. When searching for relevant papers across databases, the three categories of search terms will be connected with AND (because all of them need to be present according to our inclusion criteria). The formulation of search terms was informed by the authors’ and the consulting librarian’s collective content expertise in relevant literatures, prior scoping and systematic reviews on related topics, and publicly available search hedges (i.e., expert-developed combinations of search terms on specific topic areas) [48,49]. For PsycINFO and PubMed, a list of subject headings specific to those respective databases was also used to identify additional potentially relevant articles. See S2 Table for a complete list of search terms and subject headings used in our search and S3 Table for the full search strategy for PsycINFO.

### Study selection

Following the search, all identified citations across databases will be collated and uploaded to Rayyan [50], a systematic review management tool. The titles and abstracts of these citations will be screened by two independent reviewers for assessment against our inclusion criteria. Each reviewer may indicate whether the article meets the inclusion criteria by indicating “include,” “maybe,” or “exclude” (reviewers will be instructed to indicate “maybe” when the information in the title and abstract is insufficient to determine its eligibility). Potentially relevant sources (i.e., those that are marked as “yes” or “maybe” during the initial screening) will be retrieved in full and imported into Rayyan. During this initial screening process, disagreements will be considered to have occurred in cases where one reviewer indicates “include” or “maybe” and the other reviewer indicates “exclude.” Disagreements will be resolved through discussion between the two reviewers. If disagreements persist, disagreements will be discussed as a team, with a final decision made by the first author (DYL). In cases where one reviewer indicates “yes” and the other “maybe,” the citation will proceed to full-text screening without the need to resolve the discrepancy at this stage in the process.

The full texts of the selected citations will be assessed in detail against the inclusion criteria by two independent reviewers, each of whom who will make an “include” or “exclude” decision. Reasons for exclusion during the full-text review stage will be recorded and reported in the scoping review. During this process, any disagreements between the two reviewers (i.e., one reviewer indicated “include” and the other “exclude”) will be resolved through discussion between the two reviewers; any persisting disagreements will be discussed as a team, with a final decision made by the first author (DYL). The results of the search and the study inclusion process will be reported in full in the final scoping review and presented in a PRISMA flow diagram [51].

To facilitate training and consistency across authors, a pilot screening exercise will take place for both the initial abstract screening and the full-text screening phases. For each phase, 15 sources will be selected for piloting and training, and these sources will be screened by all members of the research team who carry out article screening tasks. The process of searching, screening, and selecting eligible sources, as well as team discussions, may reveal potentially relevant search terms and constructs and so may result in modifications of the search strategy (e.g., search terms, concepts) during the review process. Any modifications will be documented in the final publication of this review.

### Data extraction

Data will be extracted from papers included in the scoping review by at least two independent reviewers. The data extracted will include specific details about the participants, concept, context, and study methods relevant to the review questions (see S4 Table for a draft data extraction form). To test the feasibility of the planned data extraction process and facilitate training and consistency across the team, a pilot data extraction exercise will be carried out. Specifically, research team members who will participate in data extraction will be paired, and each pair will be assigned one quantitative and one qualitative study for data extraction. They will then meet to compare their data extraction sheets. The team will then meet as a group to discuss the pilot data extraction exercise and its feasibility and decide on any necessary modifications or refinements to the data extraction process. Any modifications made during the data extraction process will be reported in the final publication of this review. When appropriate, authors of papers will be contacted to request missing or additional data. Similar to the screening stage, any disagreements (i.e., discrepancies between the data extracted) on data extraction that arise between the reviewers will be resolved through discussion between the reviewers; if disagreements persist, disagreements will be discussed as a team, with a final decision made by the first author (DYL). Formal assessment of study quality and risk of bias will not be performed as these are outside the scope of the scoping review [45].

### Data analysis and presentation

For each research question, we plan to conduct basic frequency analyses and present relevant descriptive data in tables. In line with the review objective and research questions, we plan to include tables summarizing (1) different types of research questions related to affect regulation in the context of minority stress (e.g., grouping different research questions based on common themes and presenting descriptive frequencies of those themes); (2) frequencies of different types of research designs and analytic approaches used in these studies (e.g., the number of studies that used quantitative, qualitative, and mixed methods); (3) frequencies of different ways researchers have assessed affect regulation in the minority stress context (e.g., the number of studies that used global self-report surveys, daily diaries, qualitative interviews, etc.; the number of studies that used generic measures of affect regulation versus measures specifically designed to address SGM experiences), and (4) the extent to which researchers explicitly attended to the experiences of SGM subgroups or to intersecting identities characteristics (i.e., identifying different strategies that researchers used to examine subgroup experiences or intersecting identity characteristics, followed by descriptive frequencies for those strategies). Presentation of data for each research question will be followed by a narrative summary of main findings and takeaways, as well as gaps in the literature [52].

### Study timeline

At the time of registering this protocol on OSF (July 11, 2025), the first author (DYL) had conducted an initial literature search (on June 19, 2025) from the following databases: PsycINFO (via EBSCO), Web of Science Core Collection (via Clarivate), PubMed (via National Library of Medicine), Gender Studies Database (via EBSCO), Sociological Abstracts (via ProQuest), SocIndex with Full Text (via EBSCO). Downloaded citations were then uploaded to Rayyan and duplicates were removed by the first author (completed on July 5, 2025). The scoping review research team (i.e., authors of this protocol and additional undergraduate-level and post-baccalaureate research assistants) met weekly from May to July, 2025 to develop the protocol and pilot the title and abstract screening procedures. Title and abstract screening via Rayyan started on July 9, 2025.

We anticipate that the next steps of this scoping review will take approximately 24 months to complete. Specifically, the following expected timeline associated with each stage is: (a) title and abstract screening (6 months), (b) full-text screening (6 months), (c) data extraction (6 months), (d) data organization and synthesis (3 months), and (e) manuscript writing (3 months). Planned search of the grey literature will take place for the next four months, and all citations are expected to be screened by the time the full-text screening stage is complete.

## Supporting information

S1 Table Completed checklist recommended items to address in a scoping review. Adapted from the PRISMA-P (Preferred Reporting Items for Systematic review and Meta-Analysis Protocols) 2015 checklist.
(DOCX)

S2 Table Complete list of search terms and subject headings. (DOCX)

S3 Table Full search strategy for PsycINFO (via EBSCO). (DOCX)

S4 Table Draft data extraction form. (DOCX)

## References

[1] American Psychological Association. A guide to sexual orientation and gender diversity terms [Internet]. 2022 [cited 2025 Jul 10]. Available from: https://www.apa.org/ed/precollege/psychology-teacher-network/introductory-psychology/diversity-terms

[2] Association for Behavioral and Cognitive Therapies. Sexual and gender minority populations [Internet]. 2025 [cited 2025 Jul 10]. Available from: https://www.abct.org/fact-sheets/sexual-and-gender-minority-populations/

[3] American Psychological Association. Guidelines for psychological practice with transgender and gender nonconforming people. Am Psychol. 2015;70(9):832–64. doi: 10.1037/a0039906 26653312

[4] Plöderl M, Tremblay P. Mental health of sexual minorities. A systematic review. Int Rev Psychiatry. 2015;27(5):367–85. doi: 10.3109/09540261.2015.1083949 26552495

[5] Valentine SE, Shipherd JC. A systematic review of social stress and mental health among transgender and gender non-conforming people in the United States. Clin Psychol Rev. 2018;66:24–38. doi: 10.1016/j.cpr.2018.03.003 29627104 PMC6663089

[6] Brooks VR. Minority stress and lesbian women. Lexington Books. 1981.

[7] Hendricks ML, Testa RJ. A conceptual framework for clinical work with transgender and gender nonconforming clients: An adaptation of the minority stress model. Prof Psychol Res Pr. 2012;43.

[8] Meyer IH. Prejudice, social stress, and mental health in lesbian, gay, and bisexual populations: conceptual issues and research evidence. Psychol Bull. 2003;129(5):674–97. doi: 10.1037/0033-2909.129.5.674 12956539 PMC2072932

[9] Meyer IH. Minority stress and mental health in gay men. J Health Soc Behav. 1995;36(1):38–56. doi: 10.2307/2137286 7738327

[10] Testa RJ, Habarth J, Peta J, Balsam K, Bockting W. Development of the Gender Minority Stress and Resilience Measure. Psychol Sex Orientat Gend Divers. 2015;2:65–77.

[11] Hatzenbuehler ML. How does sexual minority stigma “get under the skin”? A psychological mediation framework. Psychol Bull. 2009;135(5):707–30. doi: 10.1037/a0016441 19702379 PMC2789474

[12] Diamond LM, Alley J. Rethinking minority stress: A social safety perspective on the health effects of stigma in sexually-diverse and gender-diverse populations. Neurosci Biobehav Rev. 2022;138:104720. doi: 10.1016/j.neubiorev.2022.104720 35662651

[13] Chaudoir SR, Wang K, Pachankis JE. What reduces sexual minority stress? A review of the intervention “toolkit”. J Soc Issues. 2017;73(3):586–617. doi: 10.1111/josi.12233 29170566 PMC5695701

[14] Major B, O’Brien LT. The social psychology of stigma. Annu Rev Psychol. 2005;56:393–421. doi: 10.1146/annurev.psych.56.091103.070137 15709941

[15] Lazarus RS, Folkman S. Stress, appraisal, and coping. Springer. 1984.

[16] Gross JJ. The Emerging Field of Emotion Regulation: An Integrative Review. Review of General Psychology. 1998;2(3):271–99. doi: 10.1037/1089-2680.2.3.271

[17] Troy AS, Willroth EC, Shallcross AJ, Giuliani NR, Gross JJ, Mauss IB. Psychological Resilience: An Affect-Regulation Framework. Annu Rev Psychol. 2023;74:547–76. doi: 10.1146/annurev-psych-020122-041854 36103999 PMC12009612

[18] Trudel-Fitzgerald C, Boucher G, Morin C, Mondragon P, Guimond A-J, Nishimi K, et al. Coping and emotion regulation: A conceptual and measurement scoping review. Can Psychol. 2024;65(3):149–62. doi: 10.1037/cap0000377 39371329 PMC11449430

[19] Brooks VR. Minority stress and lesbian women. Lexington Books; 1981.

[20] Diamond LM. New paradigms for research on heterosexual and sexual-minority development. J Clin Child Adolesc Psychol. 2003;32(4):490–8. doi: 10.1207/S15374424JCCP3204_1 14710457

[21] Pachankis JE. The psychological implications of concealing a stigma: a cognitive-affective-behavioral model. Psychol Bull. 2007;133(2):328–45. doi: 10.1037/0033-2909.133.2.328 17338603

[22] Miller CT, Major B. Coping with stigma and prejudice. In: Heatherton TF, Kleck RE, Hebl MR, Hull JG, editors. The Social Psychology of Stigma. New York, NY: The Guilford Press. 2000. p. 243–72.

[23] Hatzenbuehler ML, McLaughlin KA, Nolen-Hoeksema S. Emotion regulation and internalizing symptoms in a longitudinal study of sexual minority and heterosexual adolescents. J Child Psychol Psychiatry. 2008;49(12):1270–8. doi: 10.1111/j.1469-7610.2008.01924.x 18564066 PMC2881586

[24] Lattanner MR, Pachankis JE, Hatzenbuehler ML. Mechanisms linking distal minority stress and depressive symptoms in a longitudinal, population-based study of gay and bisexual men: A test and extension of the psychological mediation framework. J Consult Clin Psychol. 2022;90(8):638–46. doi: 10.1037/ccp0000749 36066865 PMC9896512

[25] Burger J, Pachankis JE. State of the Science: LGBTQ-Affirmative Psychotherapy. Behav Ther. 2024;55(6):1318–34. doi: 10.1016/j.beth.2024.02.011 39443068

[26] Lelutiu-Weinberger C, Filimon ML, Chiaramonte D, Leonard SI, Dogaru B, Pana E. A pilot trial of an LGBTQ-affirmative cognitive-behavioral therapy for transgender and gender expansive individuals’ mental, behavioral, and sexual health. Behav Ther. 2024. 10.1016/j.beth.2024.10.005 PMC12181672 40541374

[27] Pachankis J, Chiaramonte D, Scheer JR, Ankrum H, Eisenstadt B, Hobbs R, et al. Randomised controlled trial of LGBTQ-affirmative cognitive-behavioural therapy for sexual minority women’s minority stress, mental health and hazardous drinking: Project EQuIP protocol. BMJ Open. 2025;15(3):e086738. doi: 10.1136/bmjopen-2024-086738 40032395 PMC11877267

[28] Craig SL, Austin A. The AFFIRM open pilot feasibility study: A brief affirmative cognitive behavioral coping skills group intervention for sexual and gender minority youth. Children and Youth Services Review. 2016;64:136–44. doi: 10.1016/j.childyouth.2016.02.022

[29] Gross JJ. Emotion regulation: Past, present, future. Cogn Emot. 1999;13:551–73.

[30] Gross JJ. Emotion regulation: Current status and future prospects. Psychol Inq. 2015;26:1–26.

[31] Compas BE, Jaser SS, Dunbar JP, Watson KH, Bettis AH, Gruhn MA, et al. Coping and Emotion Regulation from Childhood to Early Adulthood: Points of Convergence and Divergence. Aust J Psychol. 2014;66(2):71–81. doi: 10.1111/ajpy.12043 24895462 PMC4038902

[32] Baziliansky S, Cohen M. Emotion regulation and psychological distress in cancer survivors: A systematic review and meta-analysis. Stress Health. 2021;37(1):3–18. doi: 10.1002/smi.2972 32720741

[33] Morris N, Moghaddam N, Tickle A, Biswas S. The relationship between coping style and psychological distress in people with head and neck cancer: A systematic review. Psychooncology. 2018;27(3):734–47. doi: 10.1002/pon.4509 28748624

[34] Goldbach JT, Gibbs JJ. Strategies employed by sexual minority adolescents to cope with minority stress. Psychol Sex Orientat Gend Divers. 2015;2(3):297–306. doi: 10.1037/sgd0000124 26634221 PMC4663988

[35] Dyar C. Prospective examination of mechanisms linking minority stress and anxious/depressed affect at the event level: The roles of emotion regulation strategies and proximal minority stressors. J Psychopathol Clin Sci. 2024;133(2):178–91. doi: 10.1037/abn0000882 38095971 PMC10842229

[36] Dyar C, Herry E, Pirog S. Emotion regulation strategies and coping self-efficacy as moderators of daily associations between transgender and gender diverse (TGD) enacted stigma and affect among TGD young adults assigned female at birth. Soc Sci Med. 2024;358:117261. doi: 10.1016/j.socscimed.2024.117261 39178534 PMC11403871

[37] Mann AM, Naugle AE, Lieberman E. Experiential Avoidance and Emotion Dysregulation as Mediators in the LGBTQ Minority Stress Model. Arch Sex Behav. 2022;51(7):3443–56. doi: 10.1007/s10508-022-02376-7 35951198

[38] Singh A, Dandona A, Sharma V, Zaidi SZH. Minority Stress in Emotion Suppression and Mental Distress Among Sexual and Gender Minorities: A Systematic Review. Ann Neurosci. 2023;30(1):54–69. doi: 10.1177/09727531221120356 37313338 PMC10259152

[39] Cludius B, Mennin D, Ehring T. Emotion regulation as a transdiagnostic process. Emotion. 2020;20(1):37–42. doi: 10.1037/emo0000646 31961175

[40] Lazarus RS. Psychological stress and coping in adaptation and illness. Int J Psychiatry Med. 1974;5(4):321–33. doi: 10.2190/T43T-84P3-QDUR-7RTP 4618837

[41] Arksey H, O’Malley L. Scoping studies: towards a methodological framework. Int J Soc Res Methodol. 2005;8:19–32.

[42] Peters MDJ, Godfrey CM, Khalil H, McInerney P, Parker D, Soares CB. Guidance for conducting systematic scoping reviews. Int J Evid Based Healthc. 2015;13(3):141–6. doi: 10.1097/XEB.0000000000000050 26134548

[43] Peters MDJ, Godfrey C, McInerney P, Khalil H, Larsen P, Marnie C, et al. Best practice guidance and reporting items for the development of scoping review protocols. JBI Evid Synth. 2022;20(4):953–68. doi: 10.11124/JBIES-21-00242 35102103

[44] Moher D, Shamseer L, Clarke M, Ghersi D, Liberati A, Petticrew M, et al. Preferred reporting items for systematic review and meta-analysis protocols (PRISMA-P) 2015 statement. Syst Rev. 2015;4(1):1. doi: 10.1186/2046-4053-4-1 25554246 PMC4320440

[45] Peters MDJ, Marnie C, Tricco AC, Pollock D, Munn Z, Alexander L, et al. Updated methodological guidance for the conduct of scoping reviews. JBI Evid Synth. 2020;18(10):2119–26. doi: 10.11124/JBIES-20-00167 33038124

[46] Tricco AC, Lillie E, Zarin W, O’Brien KK, Colquhoun H, Levac D, et al. PRISMA Extension for Scoping Reviews (PRISMA-ScR): Checklist and Explanation. Ann Intern Med. 2018;169(7):467–73. doi: 10.7326/M18-0850 30178033

[47] Gratz KL, Roemer L. Multidimensional assessment of emotion regulation and dysregulation: Development, factor structure, and initial validation of the difficulties in emotion regulation scale. J Psychopathol Behav Assess. 2004;26:41–54.

[48] PubMed. PubMed search filters: Sexual minorities [Internet]. 2025 [cited 2025 Jun 17]. Available from: https://hsls.libguides.com/PubMed-search-filters

[49] University of Minnesota. Library resources for transgender topics: Search terms and strategies [Internet]. 2025 [cited 2025 Jun 17]. Available from: https://libguides.umn.edu/transgender_topics/search_terms

[50] Ouzzani M, Hammady H, Fedorowicz Z, Elmagarmid A. Rayyan-a web and mobile app for systematic reviews. Syst Rev. 2016;5(1):210. doi: 10.1186/s13643-016-0384-4 27919275 PMC5139140

[51] Page MJ, McKenzie JE, Bossuyt PM, Boutron I, Hoffmann TC, Mulrow CD, et al. The PRISMA 2020 statement: an updated guideline for reporting systematic reviews. BMJ. 2021;372:n71. doi: 10.1136/bmj.n71 33782057 PMC8005924

[52] Miles DA. 2017.

