# Supplementary material for: Affect regulation in the context of sexual and gender minority stress: A scoping review protocol
Source: PLoS One. 2026 Jan 5;21(1):e0329531. doi: 10.1371/journal.pone.0329531 (PMC12768351; doi:10.1371/journal.pone.0329531)
Supplement: S3 Table — (DOCX) [file pone.0329531.s003.docx]

**S3 Table: Full search strategy for PsycINFO (via EBSCO)**

|  | LGBT* OR GLBT* OR “sexual and gender minorit*” OR “sexual gender minorit*” OR “sexual minorit*” OR “sexually diverse” OR “sexual diverse” OR gay* OR lesbian* OR homosexual* OR “same-sex” OR “non-heterosexual” OR “same-gender” OR bisexual* OR “bi-sexual*” OR pansexual* OR polysexual* OR demisexual OR asexual* OR queer OR “gender minorit*” OR “non-cisgender*” OR genderqueer OR “gender-queer” OR nonbinary OR “non-binary” OR agender* OR “gender-expansive” OR “gender divers*” OR genderfluid* OR “gender-fluid*” OR “two-spirit*” OR “bigender” OR “intersex” OR “gender non-confirming” OR “gender nonconfirming” OR “gender variant” OR “third gender*” OR transgender* OR transfeminine OR transmasculine OR transsexual* OR “trans man” OR “trans men” OR “trans woman” OR “trans women” OR “trans person*” OR “trans people” OR “trans youth” OR “trans adolescent*” OR “trans boy*” OR “trans girl*” OR “trans child*” OR “men who have sex with” OR “males who have sex with” OR “women who have sex with” OR “females who have sex with” OR DE “LGBTQ” OR DE “LGBTQ Rights” OR DE “LGBTQ Parents” OR DE “Asexuality” OR DE “Bisexuality” OR DE “Gender Nonbinary” OR DE “Gender Nonconforming” OR DE “Intersex” OR DE “Lesbianism” OR DE “Transgender” |
| --- | --- |
|  | DE “LGBTQ” OR DE “LGBTQ Rights” OR DE “LGBTQ Parents” OR DE “Asexuality” OR DE “Bisexuality” OR DE “Gender Nonbinary” OR DE “Gender Nonconforming” OR DE “Intersex” OR DE “Lesbianism” OR DE “Transgender” OR DE “Transgender (Attitudes Toward)” OR DE “Homosexuality” OR DE “Homosexuality (Attitudes Toward)” OR DE “Male Homosexuality” OR DE “Female Homosexuality” OR DE “Sexual Minority Groups” OR DE “Gender Identity” OR DE “Sexual Identity” OR DE “Pansexuality” OR DE “Same Sex Marriage” OR DE “Same Sex Couples” OR DE “Transsexualism” |
|  | S1 OR S2 |
|  | cope OR coping OR coped OR “emotion regulat*” OR “emotion regulatory” OR “emotion dysregulation” OR “regulate emotion*” OR “regulating emotion*” OR “regulated emotion*” OR “affect regulat*” OR “affect regulatory” OR “affect dysregulation” OR “regulate affect*” OR “regulating affect*” OR “regulated affect*” OR “emotion management” OR “manage emotion*” OR “managing emotion*” OR “mood regulation” OR “mood regulatory” OR “regulat* mood” OR “stress regulation” OR “regulate stress*” OR “regulating stress*” OR “regulated stress*” OR “stress management” OR “manage stress*” OR “managing stress*” OR “managed stress*” OR “cognitive reappraisal” OR “expressive suppression” OR “emotion* suppression” OR ruminati* OR distraction |
|  | DE “Affect Regulation” OR DE “Emotion Regulation” OR DE “Emotional Regulation” OR DE “Stress and Coping Measures” OR DE “Coping Behavior” OR DE “Coping Style” OR DE “Stress Management” OR DE “Expressive Suppression” OR DE “Emotional Control” OR DE “Anger Control” OR DE “Self-Regulation” OR DE “Cognitive Appraisal” OR DE “Cognitive Control” OR DE “Thought Suppression” OR DE “Suppression (Defense Mechanism)” |
|  | S4 OR S5 |
|  | “minority stress*” OR discriminat* OR violence OR mistreat* OR oppress* OR marginalize* OR microaggress* OR victimi* OR reject* OR stigma* OR prejudic* OR heterosexis* OR homonegativ* OR harass* OR homophobi* OR transphobi* OR queerphobic*, biphobi*, OR cisgenderis* OR “cis-genderis*” OR cisheterosexis* OR “cis-heterosexis*” OR cisnormativ* OR “cis-normativ*” OR heteronormativ* OR “hetero-normativ*” OR “hypervigilan*” OR “rejection sensitiv*” OR “gender dysphori*” OR “anti-LGBT*” |
|  | DE “Minority Stress” OR DE “Marginalization” OR DE “Prejudice” OR DE “Stigma” OR DE “Victimization” OR DE “Discrimination” OR DE “Sex Discrimination” OR DE “Social Discrimination” OR DE “Microaggression” OR DE “Gender Dysphoria” OR DE “Harassment” OR DE “Hate Crimes” OR DE “Hate” OR DE “Aggressive Behavior” OR DE “Oppression” |
|  | S7 OR S8 |
|  | S3 AND S6 AND S9 |
|  | Limited results of S10 to English language and applied the “apply equivalent subjects” expander |

*Note.* Used “All Fields” default option for 3, 6, and 9.
