# Supplementary material for: Affect regulation in the context of sexual and gender minority stress: A scoping review protocol
Source: PLoS One. 2026 Jan 5;21(1):e0329531. doi: 10.1371/journal.pone.0329531 (PMC12768351; doi:10.1371/journal.pone.0329531)
Supplement: S4 Table — (DOCX) [file pone.0329531.s004.docx]

**S4 Table: Draft data extraction form**

| **Basic Study Information** | |
| --- | --- |
| Authors |  |
| Year |  |
| Country in which the study was conducted |  |
| Main research questions |  |
| Hypotheses |  |
| Publication status |  |
| **Population (code by study)** | |
| Target sample | (include the authors’ descriptions) |
| Recruitment methods |  |
| Geographical location/range participants were recruited |  |
| Total sample size |  |
| Age (M, SD, and range) |  |
| Sample size breakdown of different sexual orientations |  |
| Sample size breakdown of different gender identities |  |
| Sample size breakdown of different races and ethnicities |  |
| Socioeconomic status | (e.g., income, education, employment, subject measures; use the authors’ language) |
| Other information on participants’ identities important to the paper |  |
| **Study Design (code by study)** | |
| Cross-sectional or longitudinal |  |
| (If longitudinal) Timing and frequency of follow-up(s) |  |
| Quantitative, qualitative, or mixed methods |  |
| experimental, quasi-experimental, or descriptive |  |
| Affect regulation construct(s) assessed |  |
| Methods used to assess affect regulation | (e.g., global self-report surveys, daily diaries, qualitative interviews) |
| Specific measures used to assess affect regulation | (name and citation of measures; note any measure adaptations if present) |
| Setting in which affect regulation was assessed | (e.g., research lab, naturalistic, setting participants chose to complete the survey/interview/etc.) |
| In what way did the researchers studied affect regulation *specifically in the context of minority stress* | (e.g., explicitly mentioning minority stress in research questions, experimentally inducing minority stress context, mediation analysis, moderation analysis) |
| Did the authors explicitly attend to unique experiences of subgroups of SGM people? | (If so, specify in what ways: through recruiting specific SGM subgroup(s), through measuring unique experiences of the SGM subgroup(s) measures, through statistical analysis examining subgroup differences, etc.) |
| Did the authors explicitly attend to intersecting identities of SGM people? | (If so, specify in what ways: through recruiting populations with intersecting identities, through measures, through statistical analysis, etc.) |

*Note.* This draft data extraction form will be further refined during the screening and data extraction pilot stages as we learn more about the contents of the included studies. The final data extraction form will be included in the publication of this review.
